# Male suicide rates in German prisons and the role of citizenship

**DOI:** 10.1371/journal.pone.0178959

**Published:** 2017-06-07

**Authors:** Daniel Radeloff, Thomas Lempp, Mattias Kettner, Amna Rauf, Katharina Bennefeld-Kersten, Christine M. Freitag

**Affiliations:** 1 Department of Child and Adolescent Psychiatry, Psychosomatics and Psychotherapy, Goethe University Frankfurt, Frankfurt, Germany; 2 Department of Child and Adolescent Psychiatry, Psychotherapy and Psychosomatics, University Hospital Leipzig, Leipzig, Germany; 3 Institute of Forensic Medicine, Goethe University Frankfurt, Frankfurt, Germany; 4 Institute for Suicide Research, Restorf, Hoehbeck, Germany; Universite de Bretagne Occidentale, FRANCE

## Abstract

**Purpose:**

Prisoners are at a particularly high risk of suicide. In contrast to other psychosocial risk factors it remains unclear to what degree the risk of suicide differs between prisoners with local citizenship and foreigners. In order to provide more detailed information for suicide prevention in prisons, this study aims to compare suicide rates (SR) between these populations in German criminal custody.

**Methods:**

Based on a German national database of completed suicide in custody, suicides by prisoners were analysed and compared with epidemiological data of the prison population and the general population, stratified for German and foreign citizenship. Data analysis was adjusted for differences in the age distribution of both populations by calculating standard mortality ratios (SMR) for suicide.

**Results:**

SR were higher in prisoners with German citizenship than those with foreign citizenship (SR = 76.5 vs. SR = 42.8, P<0.01). This association was not specific to the prison population, as the higher SR in citizens compared to non-citizens (SR = 19.3 vs. SR = 9.0, P<0.01) were also found in the general population. The association between prison suicide and citizenship was comparable in juvenile and adult prisoners, indicating its relevance to both the juvenile and adult detention systems.

**Conclusion:**

Imprisonment is associated with a substantially increased risk of suicide in both German and non-German citizens, a finding which needs to be taken into consideration by the justice system. The lower suicide risk in non-German citizens is independent of whether or not they are in custody.

## Introduction

Suicide continues to be a major public health concern, and worldwide more than 800,000 people die by suicide every year. It is the second leading cause of death in the 15- to 29-year-old age group [1,2]. Individuals with a mental disorder are at a particular risk of suicide, with about 90% of all suicides in high-income countries being committed by mentally ill patients [3]. According to WHO guidelines, the identification of high-risk groups is crucial in public health suicide prevention approaches, as well as ensuring that these populations receive adequate psychiatric support [4].

The association between delinquent behaviour and suicide has been shown in several large epidemiological studies and reviews [5–9]. Prisoners, i.e. persons committing an extreme form of delinquency, are at a particularly high risk of suicide. Two national surveys reported a 5-fold and 6-fold [10,11] higher risk for prisoners compared to the general population in Great Britain and Germany, respectively. The highest suicide risks were reported for adolescent prisoners, and were 18 to 23 times greater than in the general population [10,12]. Thus, prisoners clearly constitute a high-risk group for suicide.

Various factors may contribute to the elevated suicide rates (SR) in prisoners: first, they often show behaviour and personality traits (e.g. psychiatric disorders, substance abuse, history of prior suicide attempts) associated with an increased risk of suicide, even before serving a prison sentence. Second, in addition to these individual risk factors, the prison environment itself may contribute to an elevated suicide risk [13–15]. Stress-related circumstances due to confinement include separation from family and friends, violent confrontations between inmates, loss of control, heteronomy, the failure of a previous lifestyle and the ensuing phase associated with feelings of guilt and shame, as well as a loss of perspective for the future, are all contributing factors to increased SR [16–18].

The sociocultural background of the German prison population is highly heterogeneous [19]. Although sociocultural factors have a strong influence on suicide characteristics [20,21] and migration contributes to the heterogeneity of suicide risk [22], the degree to which suicide risk differs between detainees with German vs. non-German citizenship has not been studied. SR in Italian / Austrian prison populations were lower in individuals with foreign compared to local citizenship [23,24]. Since other studies have also found lower SR in non-citizens compared to citizens in the general population, this finding might not be specific to the prison environment [22,25,26]. This study analyses the association between suicide and citizenship in German inmates in comparison to the general population.

Here, we aim to address the following hypotheses: (1) SR in male prisoners are higher in citizens compared to non-citizens. (2) We expect that these differences are not prison-specific: we thus hypothesize that also in the general population, SR are higher in the population with German compared to those with foreign citizenship.

## Methods

### German criminal law and the prison system

According to German law, the age of criminal responsibility is 14 years, and there is a specific criminal law relating to young offenders aged between 14 and 18 years. Between 18 and 21 years of age, the courts have the provision to convict offenders according to the juvenile criminal law depending on emotional, mental, and intellectual maturity [27]. Individuals who are 21 years old or older are considered to be fully criminally responsible irrespective of possible maturational lags, and are usually detained in adult prisons.

### Data acquisition

We have previously reported data on suicide in German criminal and pre-trial detention [12]. The current study focuses on individuals in criminal custody only, which is a subset of the above mentioned study cohort. Only in this subset is information available on inmates’ nationality.

This study included all cases of suicide committed by males in German criminal custody between 2000 and 2013 (survey period: 14 years). To calculate SR and standardized mortality rates (SMR), the following data were collected: (1) the absolute number of suicides, and (2) the absolute number of persons in prison; (3) the absolute number of suicides, and (4) the absolute number of persons in the general population. Males represent approximately 95% of the population in German prisons. As the number of suicides in females was too low for statistical analysis after stratification, this study was limited to male prison suicide.

Data were obtained for comparable age strata (age bandings were defined as 14 to 17 years, 18 to 20 years, 21 to 24 years, 25 to 29 years, 30 to 39 years, 40 to 49 years, and 50 to 59 years; the age group 60+ was excluded since the age distribution in prison populations differs extensively from the elderly general population).

Number of suicides: In Germany, every state is obliged to report all cases of suicide in prison. On a national level, data on suicides were collected by one of the co-authors (KBK) by retrospectively analysing the *reports of unusual events* of all criminal detention centres in Germany for the given time period.

Number of persons in prison: Demographic characteristics of prisoners in Germany were provided by the annual census of the Federal Statistical Office of Germany. The number of inmates was used to calculate the number of life years (LY) spent in criminal custody during the study period. The total amount of LY represents the accumulated time at risk of the prison population. In this study, we compared suicides in relation to LY inside vs. outside of criminal custody between 2000 and 2013.

Number of suicides and the number of persons in the general population: General population annual suicides and annual population census data were obtained from the Research Data Center of the Federal Statistical Office of Germany (*Forschungsdatenzentrum der Statistischen Landesämter*, Düsseldorf, Germany, www.forschungsdatenzentrum.de).

### Citizenship

After eight years of legal residence in Germany, non-Germans have the right to become naturalised, with shorter minimum periods of residency for spouses. Children born in Germany to non-German parents automatically acquire German citizenship if one parent has been a legal resident in Germany for at least eight years [28]. Those who grew up in Germany, or whose foreign citizenship is that of an EU member state or Switzerland, may keep both their German and their foreign citizenship (dual / multiple citizenship) [29]. For reason of available data structure, in this study, individuals with German citizenship were considered to be German, regardless of the type of citizenship (single or dual / multiple citizenship) and compared to individuals without German citizenship. Information on citizenship was given in a dichotomous format (German or non-German).

### Statistical methods

Data were analysed using R (Version 3.2.3, URL: http://www.R-project.org; [30]). Suicides inside and outside prison, as well as the number of LY spent inside and outside prison, were given in 2x2 tables stratified according to age and nationality. SR (SR = number of suicides / number of 100,000 LY) were calculated for both the imprisoned population and the general population, and stratified for age and citizenship. Each 2x2 table was tested for an association between suicide and citizenship in both the prison population and the general population, and in each age group using chi-squared and Fisher’s exact tests. Pairs of 2x2 tables (prison population vs. general population) were tested for differences in the association between suicide and citizenship between populations using Tarone’s test for heterogeneity. This test was also used to compare the impact of citizenship on suicide in adolescents vs. adult detainees. To reject the null hypothesis, alpha was set to 0.05.

**Fig 1:**
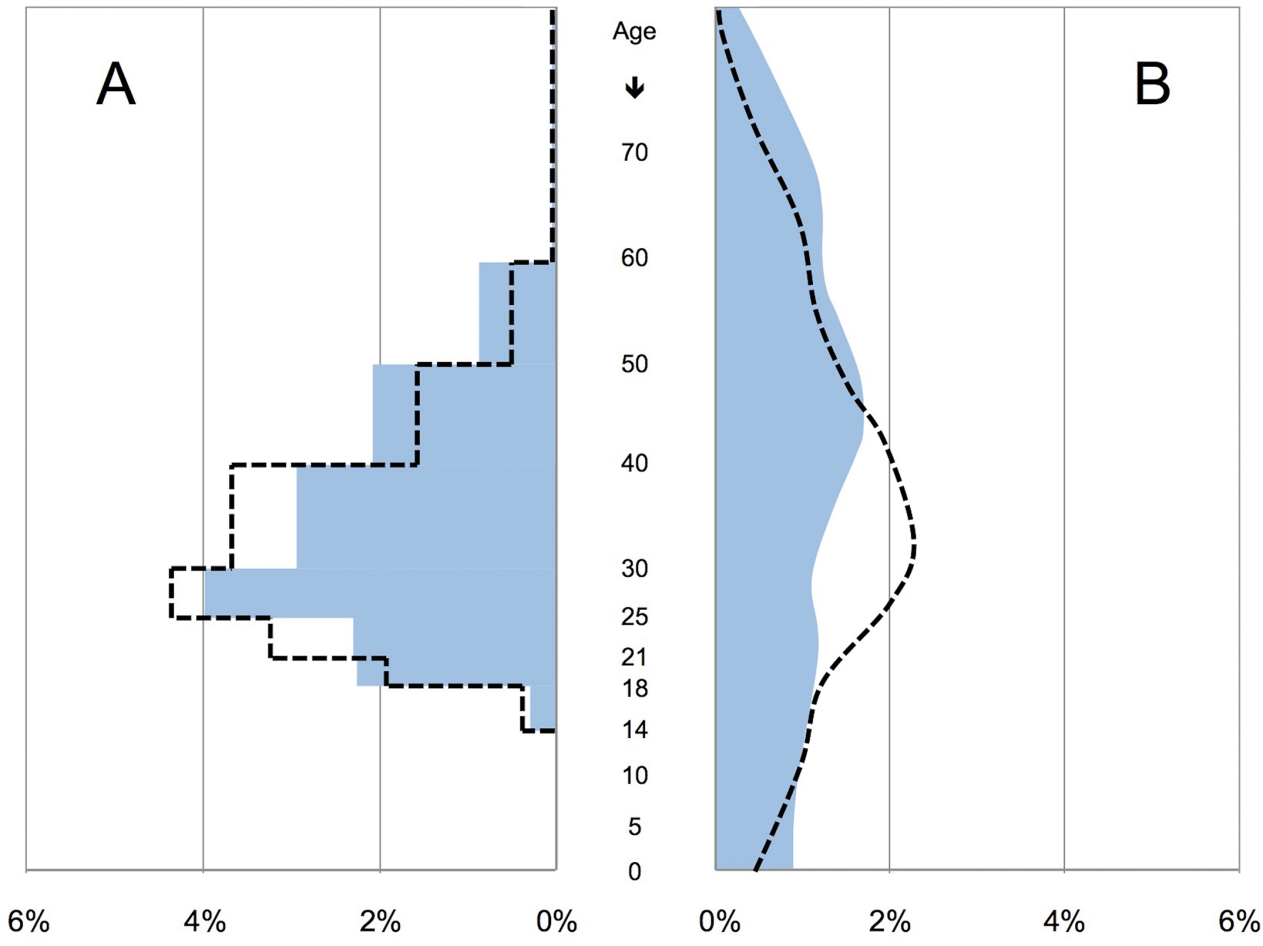
Age distribution of German and non-German citizenship. Distribution of the prison population (A) and the general population (B) stratified by German (blue) and non-German (black dashed line) citizenship. For all four populations, the total population size was defined as 100%. In the prison population, life years were given in age intervals, producing a staircase shaped graph. Data representing the general population were given for each age year.

Since the age distribution in custody differs from the general population (Fig 1), standard mortality ratios (SMR) were calculated based on age bandings defined in the census data, separately for the German and the non-German population. The SMR quantifies the increase (or decrease) in mortality of a study population compared to a reference population by taking different age distributions in both populations into account. Expected suicides (ED, expected deaths) for each age group were calculated by multiplying age-specific SR of the general population by the number of LY in detention for the specific age group. The total sum of expected suicides over all age groups was calculated and compared with the total sum of observed suicides (OD, observed deaths) in the reference population. Confidence Intervals (CI) were calculated using an approximation with CI = SMR +/- (1.96 x SE) with SE = standard error = SMR / SQR (OD) [31].

### Ethics

The study was approved by the ethics committee of the medical faculty of Goethe University Frankfurt, Germany and conducted according to the Declaration of Helsinki.

## Results

### Life years spent in prison

During the study period of 14 years, 626,452 LY were spent by male prisoners in German criminal custody, 465,424 LY of these were spent by prisoners with German citizenship, and 161,028 LY were spent by prisoners with foreign citizenship.

Distribution of age groups for German prisoners was as follows: age 14–17 (6,374 LY; 1.4%), age 18–20 (33,167 LY; 7.1%), age 21–24 (44,814 LY; 9.6%), age 25–29 (95,046 LY; 20.4%), age 30–39 (142,771 LY; 30.7%), age 40–49 (99,870 LY; 21.5%), age 50–59 (43,382 LY; 9.3%). For prisoners with foreign citizenship the distribution of age groups was as follows: age 14–17 (N = 2,370 LY, 1.5%), age 18–20 (N = 9,344 LY; 5.8%), age 21–24 (N = 20,969 LY; 13.0%), age 25–29 (N = 35,442 LY; 22.0%), age 30–39 (N = 59,730 LY; 37.1%), age 40–49 (N = 25,494 LY; 15.8%), age 50–59 (N = 7,679 LY; 4.8%). Age distribution in prison differs extensively from age distribution in the general population in both German and non-German citizens (Fig 1).

### Suicides in prison

During the study period, 425 prisoners died by suicide in German criminal custody; 356 were German and 69 were foreign citizens. Detailed data on suicides and SR in different age groups are presented in Table 1.

**Table 1:** Suicide rates of citizens and non-citizens within the prison population and the general population.

Age	Prison population						P
	German citizenship			Foreign citizenship			
	suicides	life years	SR	suicides	life years	SR	
14–17	6	6,374	94.1	1	2,370	42.2	
18–20	30	33,167	90.5	8	9,344	85.6	
21–24	39	44,814	87.0	6	20,969	28.6	*
25–29	52	95,046	54.7	8	35,442	22.6	*
30–39	114	142,771	79.8	35	59,730	58.6	
40–49	69	99,870	69.1	7	25,494	27.5	*
50–59	46	43,382	106.0	4	7,679	52.1	
**Total**	356	465,424	76.5	69	161,028	42.8	**
Age	General population						P
	German citizenship			Foreign citizenship			
	suicides	life years	SR	suicides	life years	SR	
14–17	1,002	22,839,209	4.4	52	2,304,992	2.3	**
18–20	2,118	17,852,912	11.9	154	1,913,213	8.1	**
21–24	3,433	24,260,371	14.2	302	3,321,560	9.1	**
25–29	4,496	29,372,173	15.3	489	5,385,717	9.1	**
30–39	12,373	69,454,877	17.8	1,053	11,468,500	9.2	**
40–49	19,836	85,294,917	23.3	833	8,556,642	9.7	**
50–59	18,298	70,296,259	26.0	598	5,923,147	10.1	**
**Total**	61,556	319,370,718	19.3	3,481	38,873,771	9.0	**

SR = suicide rate; asterisk indicates significant results with P < 0.05 (*) / P < 0.01 (**).

### Suicides of citizen vs. non-citizen prisoners

Prisoners with foreign citizenship showed significantly lower SR compared to German prisoners (total age range, OR 0.56, CI(95%) = 0.43–0.73, p < 0.001, x^2 = 19.45). This difference reached significance within age groups 21–24 years (OR 0.33, CI(95%) = 0.14–0.08, p = 0.012, x^2 = 6.24), 25–29 years (OR = 0.41, CI(95%) = 0.20–0.87, p = 0.024, x^2 = 5.12) and 40–49 years (OR 0.40, CI(95%) = 0.18–0.87, p = 0.023, x^2 = 5.14). Within the other age groups, absolute SR were lower in prisoners with foreign citizenship, but the 95% confidence interval of the OR was not below 1, reflecting the lower number of individuals in these strata.

While absolute SR in prisoners with foreign citizenship were lower compared to German prisoners, relative SR, calculated as SMR, were not significantly different. German men had a 4.2-fold increased suicide risk when exposed to the risk factor of custody, compared to age equivalent men with German citizenship in the general population (SMR_total_ = 4.2, CI(95%) = 3.7–4.5). Men with foreign citizenship had a 4.7-fold increased suicide risk compared to the general non-German population (SMR_total_ = 4.8, CI (95%) = 3.6–5.9). The SMR of different age groups are presented in Table 2.

**Table 2:** Standard mortality ratios (SMR) of citizens and non-citizens exposed to the risk factor imprisonment.

Age	German citizenship			Foreign citizenship		
	Observed suicides	Expected suicides	SMR (95% CI)	Observed suicides	Expected suicides	SMR (95% CI)
14–17	6	0.3	21.5 (4.3–38.7)	1	0.0	20.7 (-19.9–61.3)
18–20	30	3.9	7.6 (4.9–10.3)	8	0.7	11.5 (3.5–19.5)
21–24	39	6.3	6.1 (4.1–8.0)	6	1.8	3.4 (0.7–6.4)
25–29	52	14.5	3.6 (2.6–4.6)	8	3.1	2.6 (0.8–4.4)
30–39	114	25.4	4.5 (3.7–5.3)	35	5.3	6.5 (4.4–8.7)
40–49	69	23.2	3.0 (2.3–3.7)	7	2.7	2.6 (0.7–4.6)
50–59	46	11.3	4.1 (2.9–5.3)	4	0.9	4.5 (0.1–8.8)
**Total**	356	85.1	4.2 (3.7–4.5)	69	14.5	4.8 (3.6–5.9)

SMR = Standard Mortality Ratio; CI = confidence interval. The SMR quantifies the increase (or decrease) in mortality of a study population (detainees) compared to a reference population (general population) by taking different age distributions in both populations into account.

The association between prison suicide and citizenship was not significantly different between adolescent inmates (14–20 years of age; SR_citizen_ = 91.0, SR_non-citizen_ = 76.8) and adults (21–59 years of age, SR_citizen_ = 75.1, SR_non-citizen_ = 40.2; Tarone’s test for heterogeneity: x^2 = 1.329, df = 1, p = 0.249).

## Discussion

This study reports the following main results: (1) Among prisoners, SRs are higher in inmates with German citizenship (SR = 76.5) compared to detainees with foreign citizenship (SR = 42.8), (2) The association between citizenship and suicide is not specific to the prison population, as higher SRs in citizens (SR = 19.3) compared to non-citizens (SR = 9.0) were also found in the general population; (3) Relative SRs in citizens and non-citizens were roughly equal, representing a 4- to 5-fold increased suicide risk in prisoners compared to the general population in both citizens (SMR = 4.2) and non-citizens (SMR = 4.8). (4) The association between prison suicide and citizenship was not significantly different between adolescent and adult inmates, indicating its relevance in both the juvenile and adult detention systems.

In agreement with previously published datasets [12,10,32], SR were substantially elevated in the population of prisoners. This can be partly explained by the particular composition of the prison population [33].

Immigration is associated with many stress-related factors, including the breakdown of existing social ties, language barriers and adaptation to life in a new social and cultural environment. Still, this study showed that the suicide risk of prisoners with foreign citizenship was nearly half that of German inmates. This is in accordance with studies conducted in other countries: an Austrian study examined prison suicides (N = 220) and found higher SR in Austrian prisoners (SR = 172.2) compared to non-Austrians (SR = 106.6) [24]; an Italian study showed a doubled suicide risk (SR = 120.0) in Italian compared to non-Italian convicts [23]. In contrast, prevalence rates of attempted suicides or self-harm (both risk factors for completed suicide) were not elevated in prisoners with Italian citizenship compared to those with non-Italian citizenship [23], suggesting that results of our study cannot be transferred to suicide attempts or non-suicidal self-harm.

This study found lower SRs in non-citizens, not only in the prison population but in the general population as well. In agreement with this, lower SR were reported in immigrants compared to the German general population between 1980 and 1997 [25]. Both findings indicate that the association between citizenship and suicide is not specific to the prison environment. Lower SR for foreign citizens in Germany might result from a favourable risk profile. Immigrants partly transfer their risk profile to the host country since SR in immigrant groups correlate with SR in their country of origin [26,22,34,35]. Speculative, severe mental or physical illness (e.g. severe depressive disorder, psychotic disorder, bipolar disorder) might hinder people from immigrating in the first place. Conversely, immigrant populations in Germany might have higher physical and psychiatric conditioning compared to the general population of the country of origin, and thus a lower risk profile. In a second step, these resilience profiles might be transferred to the prison environment, resulting in different SR between inmates with German and foreign citizenship.

From a European perspective, there is no generalizable risk pattern of suicide in European immigrants [22]. Suicide risk in immigrants depends to a considerable degree on regional cultural factors, and on the risk profile in the countries of origin. As a trend, suicide risk is higher in immigrants from Northern and Eastern Europe compared to the host population and lower in immigrants from Southern Europe [22]. Still, definitions of immigrant populations vary between studies, making it difficult to compare results.

The age distribution of populations with foreign citizenship differs extensively from the German population and SRs are associated with rising age. SMR were therefore calculated taking different age distributions into account. The SMR of suicide is roughly equal for both German (SMR = 4.2) and non-German males (SMR = 4.8), meaning that after adaption for age distribution of the subgroups studied, in German and non-Germans, the risk of death by suicide is 4 to 5 times higher when exposed to the risk factor of imprisonment. Hence, imprisonment has a strong impact on suicide risk in both populations. To our knowledge, this is the only study comparing natives and immigrants in prison populations with respect to the age distribution of these populations.

Scientific findings gained from adult prisons cannot be transferred directly to juvenile detention facilities since there are significant differences between the underlying criminal codes as well as between inmate composition and the types of criminal offences committed [36,19]. In contrast, studies focussing on age-dependent risk factors for prison suicide are scarce [33], leading to a limited evidence basis of risk profiles in adolescent prisoners [4]. In this study, the association between prison suicide and citizenship was not significantly different between adolescent and adult prisoners, indicating its relevance in both juvenile and adult detention.

Reasons for the differing suicide risks between German and non-German prisoners found in this study remain speculative, due to the lack of data regarding protective and risk factors. Aiming to understand the complex causality that leads to differing suicide risks between subpopulations in custody, we suggest prospective approaches with large sample sizes. Risk profiles should include the most prominent risk and protective factors (including criminological, medical, socioeconomic and environmental information) at intake, during their sentence, and after release.

## Limitations

(1) Non-citizens are a very diverse group of people with quite different sociocultural backgrounds and a very different suicidal base risk depending on country of origin. The results of our study reflect the pooled risks of immigrants in Germany. Moreover, the composition of different nationalities differs between populations in custody and in the general population. (2) This study does not provide information about why suicide rates differ between citizens and non-citizens in criminal custody. Prevalence rates of risk factors for suicide in prison (e.g. psychiatric and medical comorbidity, history of substance abuse and prior suicide attempts [37,38] should be taken into account in future prospective studies. (3) This study examined differences between non-German and German citizens. Results are not transferable to second-generation immigrants who hold dual German/non-German citizenship. (4) This study reflects the situation in Germany and is not fully transferable to other countries. (5) All studies of the epidemiology of prison suicide should acknowledge that the higher level of intramural monitoring in prisons results in suicides having a greater chance of being declared as the correct cause of death than in the general population.

## Conclusion

Imprisonment is associated with a substantially increased risk of suicide in both Germans and immigrants. Sociocultural factors are strongly related to prison suicide risk and thus need to be considered in future prevention strategies.

## Supporting information

S1 Table Detailed data on the number of suicides and individuals in the prison population and in the general population. Sheet 1—Prison Population by Nationality, Age Group, Year, and Gender. Sheet 2—Prison Suicides by Nationality, Age Group, and Gender. Sheet 3—General Population by Nationality, Age, Year, and Gender. Sheet 4 General Population by Nationality, Age, and Gender (sum of years 2000–2013). Sheet 5—Suicides in the General Population by Nationality, Age Group, and Gender.
(XLSX)
